# Supplementary material for: Differences in Medium-Induced Conformational Plasticity Presumably Underlie Different Cytotoxic Activity of Ricin and Viscumin
Source: Biomolecules. 2022 Feb 11;12(2):295. doi: 10.3390/biom12020295 (PMC8961613; doi:10.3390/biom12020295)
Supplement: Supplementary file 1 [file biomolecules-12-00295-s001.zip › biomolecules-1564702-supplementary.pdf]

Supplementary materials

Differences in medium-induced conformational plasticity presumably underlie different cytotoxic activity of ricin and viscumin

Pavel E. Volynsky[1*], Diana V. Maltseva[2*], Valentin M. Tabakmakher[1,3], Eduard V. Bocharov[1,4], Maria P. Raygorodskaya[5], Galina S. Zakharova[5], Elena V. Britikova[6], Alexander G. Tonevitsky[1,2,5], and Roman G. Efremov[1,2,4]

[1] Shemyakin-Ovchinnikov Institute of Bioorganic Chemistry RAS, Miklukho-Maklaya str. 16/10, 117997, Moscow, Russia.
[2] National Research University Higher School of Economics, Myasnitskaya ul. 20, 101000, Moscow, Russia.
[3] School of Biomedicine, Far Eastern Federal University, FEFU Campus, 10 Ajax Bay, Russky Island, Vladivostok, 690922, Russia
[4] Moscow Institute of Physics and Technology, Institutskiy per., 9, Dolgoprudny, Moscow Region, 141701, Russia.
[5] Scientific Research Center Bioclinicum, Ugreshskaya str. 2/85, 115088, Moscow, Russia
[6] Institute of Bioorganic Chemistry NASB, Kuprevich St. 5, Minsk, 220141, Minsk, Belarus

Corresponding author

Pavel E. Volynsky mailing address: 117997, Russian Federation, Moscow, GSP-7, Ulitsa Miklukho-Maklaya, 16/10; telephone number: +79167968435; fax number: +74953350812; e-mail: volynski@yandex.ru

Table S1. Simulation systems used in this work

ID	System composition	simulation details
rd	dimeric form of ricin, 30018 water molecules, 92 NA+, 90 Cl-	orthoscopic box, volume 1000 nm^3, temperature 310K, simulation time 200 ns
md	dimeric form of viscumin, 30129 water molecules, 96 NA+, 90 Cl-	orthoscopic box, volume 1000 nm^3, temperature 310K, simulation time 200 ns
w310	RTA, 21050 water molecules	dodecahedron box; volume 678 nm^3 , temperature 310K, simulation time 10 mks
w340	RTA, 21050 water molecules	dodecahedron box; volume 678 nm^3 , temperature 340K, simulation time 10 mks
u340	RTA, 3247 urea molecules, 12057 water molecules.	dodecahedron box; volume 678 nm^3 , temperature 340K, simulation time 10 mks
m340	RTA, 2797 CHCl3 molecules, 2749 MeOH molecules	dodecahedron box; volume 678 nm^3 , temperature 340K, simulation time 10 mks

Secondary structure of studied proteins.

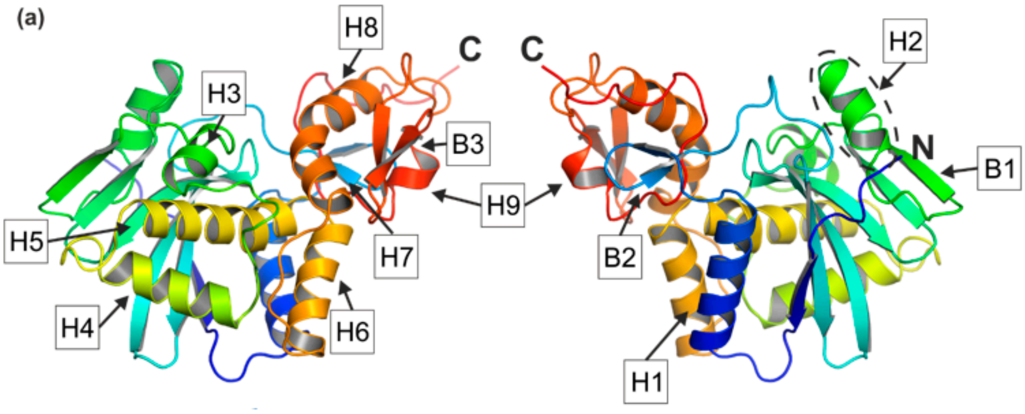

Figure S1. Ribbon representation of RTA with marked secondary structure elements (two views are given).

Table S2. Secondary structure elements of RTA and MLA

Helices	RTA a.a.	MLA a.a.
**H1**	18-32	14-27
**H2**	98-107	-
**H3**	123-129	115-122
**H4**	141-153	131-142
**H5**	161-180	148-167
**H6**	182-193	169-180
**H7**	202-208	190-195
**H8**	211-219	199-207
**H9**	246-248	233-235

Strands	RTA a.a.	MLA a.a.
**B1**	8-12;<br>57-63;<br>69-75;<br>81-86;<br>89-92;<br>114-116	3-7;<br>53-59;<br>65-71;<br>77-81;<br>86-88;<br>107-108
**B2**	38, 39, 42, 43	33, 34, 37, 38
**B3**	225-233;<br>239-244	213-221;<br>227-232

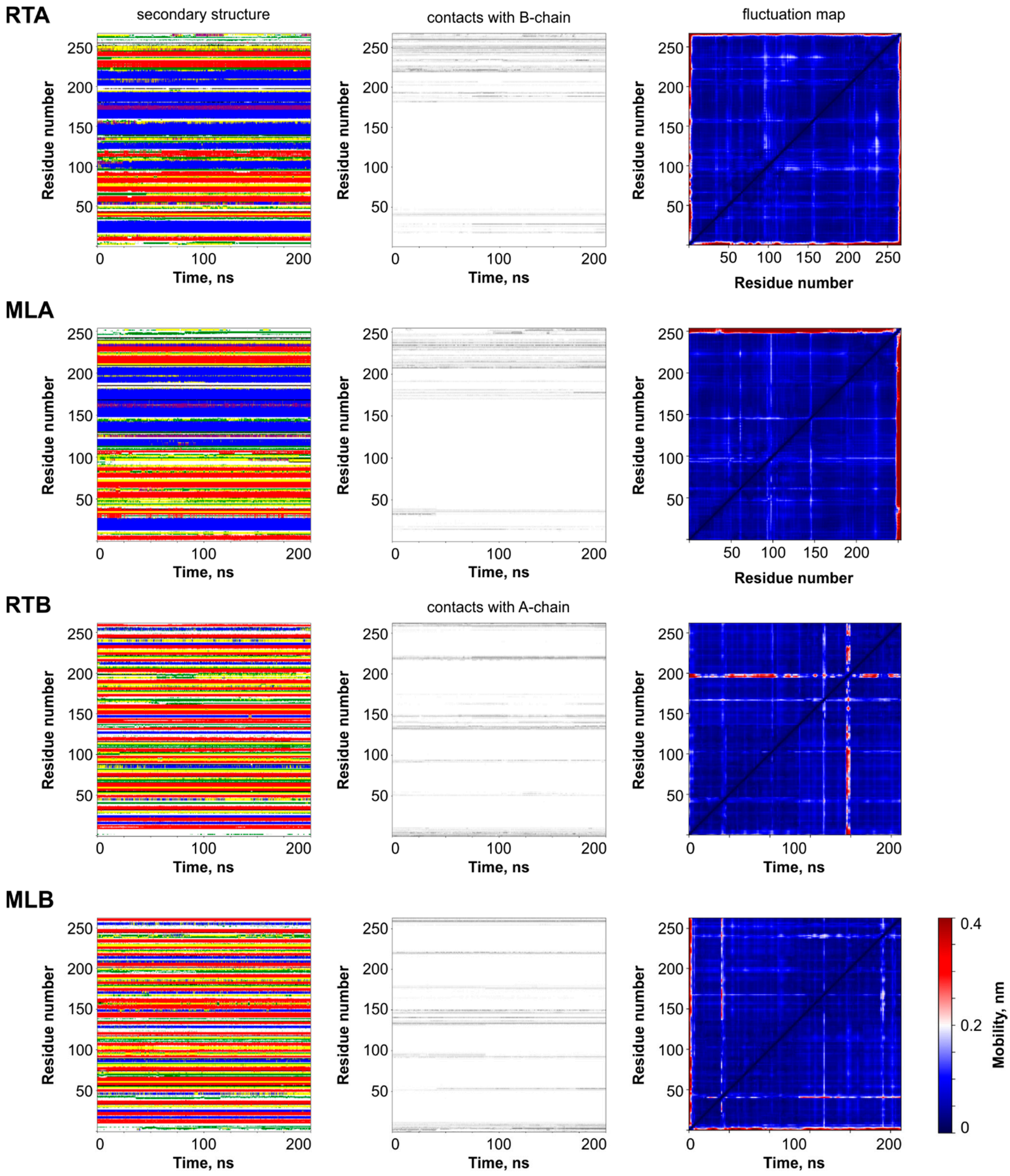

Figure S2. Results of MD simulations of full-length dimeric forms of ricin and viscumin in water at 310 K. The columns represent the evolution of the secondary structure, contacts with another subunit of the dimer, and fluctuation maps. The rows of panels (from top to bottom) correspond to the RTA, MLA, RTB, and MLB subunits. The default color code for secondary structure plots - random coil: white, β-sheet: red, β-bridge: black, bend: green, turn: yellow, α-helix: blue, π-helix: purple, 310-helix: gray. The fluctuation maps are color-coded according to the RMSF values (shown on the right).

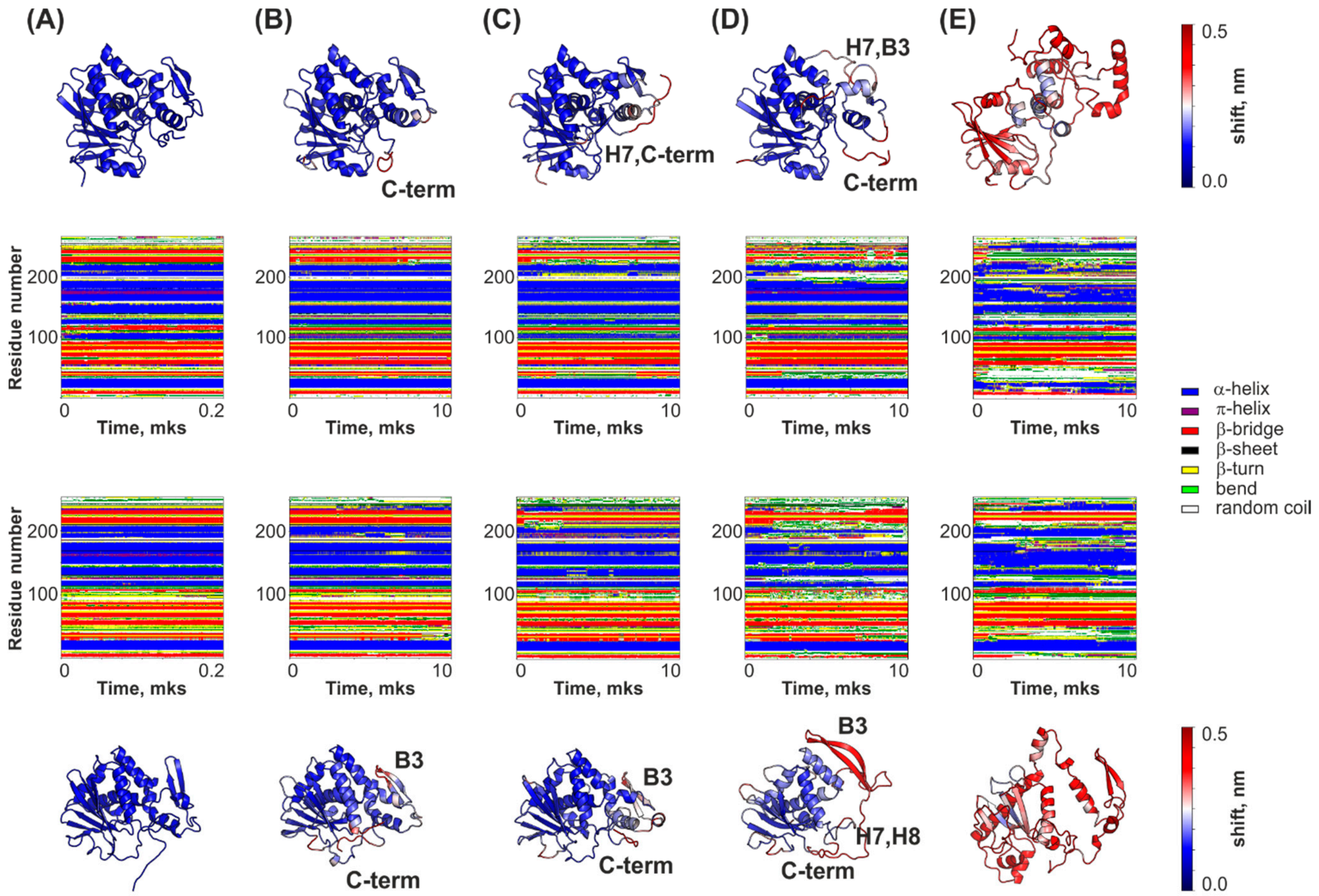

Figure S3. Solvent and temperature effects on the structure and dynamics of RTA and MLA as probed by MD simulations: Evolution of the secondary structure. Columns correspond to structural-dynamic properties of the A subunits revealed by MD simulations of the full-length toxin heterodimers in water at 310 K (A) and of the isolated A subunits in water at 310 K (B), water at 340 K (C), urea/water mixture (D), and chloroform/methanol mixture (E). Evolution of the secondary structure and the final spatial structures of RTA (two upper rows) and MLA (two lower rows) after each MD simulation are presented. Color-coding of secondary structure maps is shown in the right.

Section S1. Evolution of RTA and MLA structure in methanol-chloroform mixture.

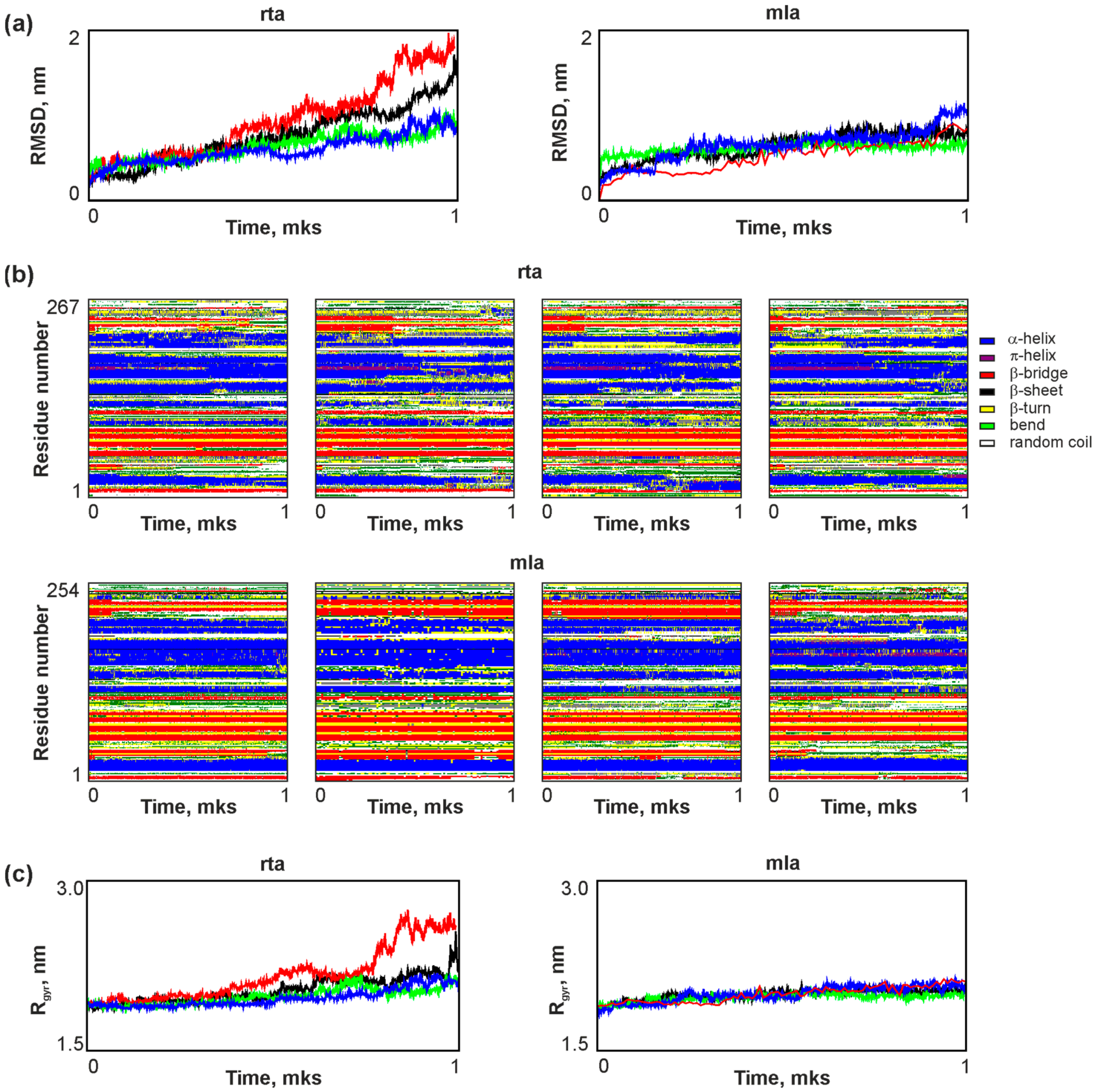

Figure S4. Evolution of RTA and MLA structure in methanol-chloroform mixture. (a) RMSD from the starting structure, calculated for 4 independent starts. The values obtained in different MD simulations are indicated by colors of the curves. (b) Evolution of the secondary structure in different MD. Color-coding of secondary structure elements are given at the right side of the maps. (c) Evolution of the proteins radius of gyration in MD simulations.

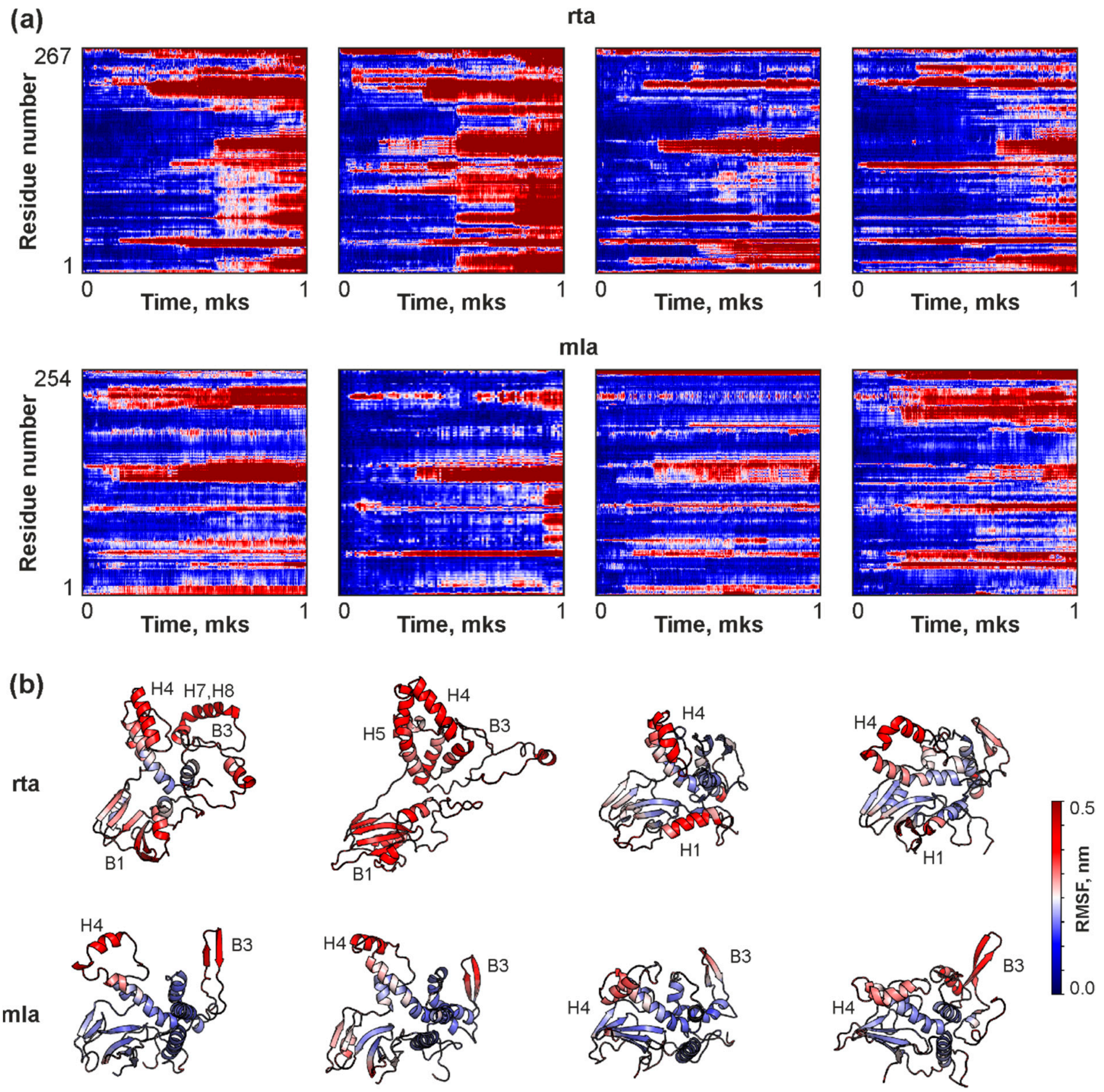

Figure S5. Evolution of RTA and MLA structure in methanol-chloroform mixture. (a) Maps of structure evolution. The values of the shift of CA atoms from its starting position after least square fitting of the structures are coded by color. (b) The structures of the RTA (upper) and the MLA (lower) after 1 mks of MD given in ribbon representation colored according to RMSF values. Secondary structure elements with large mobility are signed.

To verify the different structure evolution of studied proteins in methanol-chloroform mixture 3 additional 1 mks MD simulations were carried out. Simulations showed that similar to long MD run RTA was much plastic then MLA. The RMSD from the starting structure were higher (fig s4a). The secondary structure of proteins were stable in simulations (Fig. S4b). However, their tertiary structure has changed. The radius of gyration increased for both proteins (fig s4c). The evolutions of CA atoms drift (fig s5c) showed that RTA structure usually started its adaptation to new solvent from the region of B3 and adjacent helices H7 and H8. Loss of contact of this region with the protein nucleus leads to an increase in the size of the protein due to the loss of the core structure (fig s5b). In MLA case, adaptation went

through motion of H4 and B3 with contacted helices – other mobile regions are loops (fig s5a). The protein size were more stable (fig s4c) similar to the protein core structure (fig s5b).

The observed proteins behaviour may be explained form the analysis of the structure of the proteins core (fig s6). In RTA, B1 contacts neighboring helices (H3, H4, H5) mainly through aromatic residues, and after removing the wall (helices H7, H8), these helices can slide along B1 and easily change the core configuration. In MLA, aforementioned region contains many aliphatic residues and their relative motions are inconvenient.

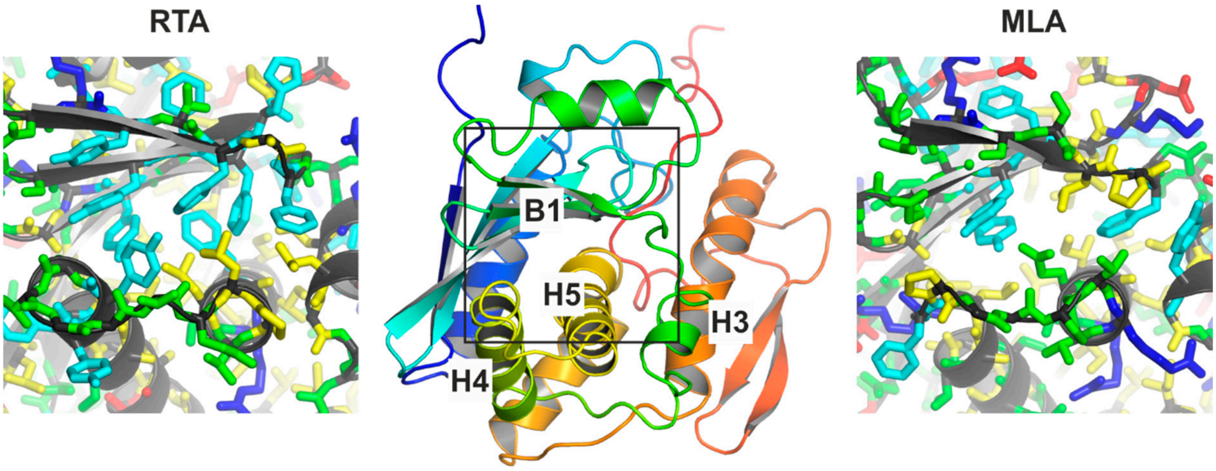

Figure s6. Organization of the hydrophobic core of RTA and MLA. The middle figure is a view of water-adapted RTA structure with the contact between B1 and adjacent helices H3, H4, H5 highlighted. Right and left panels are detailed views of the selected region in RTA (left) and MLA (right). Aromatic and non-polar aliphatic residues are shown with blue and yellow colors. Polar residues are green. In contrast to MLA, continuous stacking interactions of aromatic rings of Y84, Y91, F93, Y115, F117, F119, Y154, and F168 are observed in the hydrophobic core of RTA.

Section S2. Interaction of RTA and MLA adapted to methanol-chloroform mixture with implicit membrane.

Two prominent membrane-binding sites were found for the low-energy MC states of RTA after 5 μs MD run. In the first one, the toxin interacts with the membrane by residues 51-53, 215, 218 (the so-called “core” of the binding site), and occasionally – by residues 50, 211-214, 216, 217, 219-221 (“optional” residues). In the other site, residues of the helices H4 and H5 143, 144, 147-161, 164, 165, 167, 168, 171, 172 form the “core”, and residues 115, 118, 119, 126, 130-135 are “optional” (when these residues are involved in membrane binding, the entire RTA fragment comprising residues 143-176 is immersed into the membrane-mimicking hydrophobic slab). During subsequent 5 μs of MD simulation, both sites “evolved” due to structural perturbations although they did not disappear: for the RTA model obtained after 10 μs, two low-energy variants of interaction with the membrane were detected as well. In the site-1, the “core” residues are 2, 3, 52, 53, while residues 1, 4, 5, 32, 33, 35, 51, 54, 102, 211, 212, 214, 230, 231 are “optional”. In the other case, the corresponding residues are 143-175 and 10-16, 61-72, 84-89, 91, 257, respectively. Interestingly, that neither after 5 μs, nor after 10 μs site-1 does not overlap with the hydrophobic clusters on the surface of ricin refolded in nonpolar medium On the contrary, in both models, site-2 almost entirely falls on the large hydrophobic zone, which appears on the

RTA surface. Despite the fact that these completely restructured models of RTA and MLA have several homologous regions interacting with the hydrophobic slab (mainly in helices H6, H8), the modes of membrane binding are completely different. Accommodation of the two MLA models (after 4 and 10 μs MD) with respect to the implicit membrane revealed that the strongly unfolded toxin binds via two partially overlapping sites composed by residues 210-215, 218-246 and 128-135, 207-215, 233-245, 251, respectively. The sites are characterized by different patterns of protein insertion into the hydrophobic slab. It is evident that both models demonstrate much stronger interactions with the membrane as compared with the urea/water-adapted forms.
